# Social class related inequalities in household health expenditure and economic burden: evidence from Kerala, south India

**DOI:** 10.1186/1475-9276-10-1

**Published:** 2011-01-07

**Authors:** Subrata Mukherjee, Slim Haddad, Delampady Narayana

**Affiliations:** 1 Institute of Development Studies Kolkata Calcutta University Alipore Campus 1 Reformatory Street, 5th Floor Kolkata 700027, West Bengal, India; 2 International Health Unit CRCHUM, University of Montreal 3875 Rue Saint-Urbain, 5th Floor Montreal (Quebec), H2W 1V1, Canada; 3 Centre for Development Studies Prasanth Nagar Road, Ulloor Thiruvananthapuram 695011, Kerala, India

## Abstract

**Background:**

In the Indian context, a household's caste characteristics are most relevant for identifying its poverty and vulnerability status. Inadequate provision of public health care, the near-absence of health insurance and increasing dependence on the private health sector have impoverished the poor and the marginalised, especially the scheduled tribe population. This study examines caste-based inequalities in households' out-of-pocket health expenditure in the south Indian state of Kerala and provides evidence on the consequent financial burden inflicted upon households in different caste groups.

**Methods:**

Using data from a 2003-2004 panel survey in Kottathara Panchayat that collected detailed information on health care consumption from 543 households, we analysed inequality in per capita out-of-pocket health expenditure across castes by considering households' health care needs and types of care utilised. We used multivariate regression to measure the caste-based inequality in health expenditure. To assess health expenditure burden, we analysed households incurring high health expenses and their sources of finance for meeting health expenses.

**Results:**

The per capita health expenditures reported by four caste groups accord with their status in the caste hierarchy. This was confirmed by multivariate analysis after controlling for health care needs and influential confounders. Households with high health care needs are more disadvantaged in terms of spending on health care. Households with high health care needs are generally at higher risk of spending heavily on health care. Hospitalisation expenditure was found to have the most impoverishing impacts, especially on backward caste households.

**Conclusion:**

Caste-based inequality in household health expenditure reflects unequal access to quality health care by different caste groups. Households with high health care needs and chronic health care needs are most affected by this inequality. Households in the most marginalised castes and with high health care need require protection against impoverishing health expenditures. Special emphasis must be given to funding hospitalisation, as this expenditure puts households most at risk in terms of mobilising monetary resources. However, designing protection instruments requires deeper understanding of how the uncovered financial burden of out-patient and hospitalisation expenditure creates negative consequences and of the relative magnitude of this burden on households.

## Background

While lack of effective access to health care by marginalised groups occurs in all societies, it is more pronounced in developing countries [1,2]. Poverty, social exclusion and deprivation have a major impact on access to health care and on health [2-4]. Demand-side barriers have been found to be as important as supply-side constraints in determining populations' access to health care, especially for the poor and for vulnerable groups [5]. Moreover, in India, high levels of private health care expenditure and out-of-pocket expenditure are placing considerable financial burden on households [6].

In the Indian context, it is not easy to identify a variable that can stratify the population into subgroups for analysing inter-group inequality and disparity. Studies have used economic class, social class/caste, religion, region, gender and age group as variables to stratify the population [7]. Among them, income class and caste are often considered the most powerful stratification variables for analysing socio-economic inequality [8]. Though BPL (below poverty line) surveys have been carried out in rural India to identify poor populations, they suffer from limitations such as poor quality and low coverage, political influence, corruption and methodological flaws [9-11]. In many cases, households' caste characteristics have been found to be more appropriate for identifying poverty and vulnerability status [3]. In the Indian context, caste indicates a hereditary, endogenous, closed and immutable group having a traditional association with an occupation and a particular position in the social hierarchy, whereas socio-economic status is an open and non-immutable characteristic of a household or group of households [12,13]. Scheduled Castes (SC), Scheduled Tribes (ST) and Other Backward Castes (OBC) are considered the socially backward classes. SC and ST together account for nearly one-quarter of the population [7,14,15]. Moreover, most of the backward caste population lives in rural areas that account for 73% of the population but have only 25% of the country's health infrastructure, medical manpower and other health care resources [14,16]. The increased dependence of the population, including the poor and vulnerable, on private health care providers, the lack of health insurance, and out-of-pocket payments are leaving many impoverished [17-23].

Social inequalities in health have been analysed in various contexts, although not many studies have analysed caste-based health inequality in the Indian context and in other low-income countries [24]. The south Indian state of Kerala is widely known for its superior social and health achievements despite low levels of income [25-29]. Kerala also exhibits less inequality in health and educational achievements between social groups than is observed in other Indian states [26,30]. Nevertheless, inter-caste disparity continues to underlie overall disparity in Kerala in terms of expenditure on food and clothing, landholdings and educational levels of heads of households [7]. Though known to exist, inter-caste inequality in out-of-pocket health expenditure has not been studied in the Kerala context. This paper addresses this research gap by examining the inequalities in households' health expenditure in Kerala and its burden by caste groups to uncover the linkages between caste, poverty, effective access to health care and the burden of health expenditure.

### Study objectives

This paper has the following two broad objectives:

1. To assess the caste-based inequalities in per capita health expenditure controlling for household's health care needs and types of illness episode.

2. To assess the variation in economic burden of out-of-pocket health expenditure across different caste groups.

### Framework

In a context where health insurance is almost non-existent and the population depends on private health care providers to a large extent, households' out-of-pocket health expenditure can be a good reflection of their health care consumption and their effective access to quality health care. By analysing households' per capita health expenditure while taking into consideration their burden of health care need, we were able to identify the influence of social stratification at equal levels of need. To do this, we used a multivariate linear regression framework with the following specification:

Ln (per capita health expenditure) = f (caste, landholding, health care need, sex of the household head, health care utilisation)

In Kerala's rural society, households' education and income levels are largely determined by their socio-economic status, and social class (caste) and landholdings together can capture the socio-economic status of the household to a great extent. In informal rural economies, it is not easy to get reliable income data at the household level. A household's per capita consumption expenditure or asset holding (including landholding) is often used as a second-best proxy to capture its economic status [31]. We analysed households' economic burden of health care and variations across caste groups by identifying households that spend relatively large amounts on health care and investigating their sources of funds for out-of-pocket health care expenditures. The analysis was mostly done at the household level, except when we estimated per capita health expenditure by type of illness episode.

### Context

The context of our study was Kottathara Panchayat (panchayat being the lowest layer of the decentralised government) in the Wayanad District of India's southern state of Kerala. Wayanad has a large tribal population [14,32]. Kottathara, with a population of 16,613, is a multi-religious, multi-caste/tribe setting with 3% SC and 28% ST, predominantly Paniya and Kurichiar tribes [14]. Kottathara Panchayat has one primary health centre and four sub-centres. The district hospital in Mananthavady is 30 kilometres away, but private health care facilities, including clinics, nursing homes and hospitals, are located in Kalpetta, about 10 kilometres away.

## Methods

### Design

The major source of data used for the analysis was a 12-month long panel survey conducted between October 2003 and September 2004. The panel survey was preceded by a baseline survey in 2003 that covered the entire population of Kottathara Panchayat. The panel survey of 2,925 individuals from 543 sample households was designed to collect detailed information on health care consumption for all reported episodes. In comparison to single-visit cross sectional surveys with long recall periods, data from a year-long panel survey provide more accurate information on the incidence of illness episodes and on out-of-pocket health expenditure with a minimum level of recall bias. Details were recorded on consumption of out-patient (OP) visits, in-patient hospitalisations and expenses incurred. The total number of uncensored episodes (i.e., episodes for which we have full information) reported by the sample population was 4,949, consisting of 4,408 acute, 430 chronic and 111 pre-natal, delivery and preventive care episodes.

### Variables

#### Households' annual health care expenditure

Expenditure on health care includes all payments made to hospitals, physicians, or any other health care provider, including for dental care. It also includes expenses related to obtaining health care services, such as transportation costs to the health facility, lodging or accommodation costs (in cases where the health care facility is outside the Panchayat) and food consumed away from home during a health visit.

#### Social stratification

The official Indian classification divides all households into four broad caste categories, namely, Scheduled Tribes (ST), Scheduled Castes (SC), Other Backward Castes (OBC) and others (i.e., residual categories). For our purposes, we reclassified all castes into four hierarchical categories of social order, from lower to upper caste: Paniya (the most marginalised ST), Other ST/SC, OBC and FC (Forward Caste, at the top in the caste hierarchy). As described in the Results section, we validated our reclassification of social categories by exploring select indicators of socio-economic status across the four castes.

#### Households' health care need

We defined the level of a household's health care need based on two characteristics: the number of household members who were elderly (aged 60 years or more) and the number of members with chronic illness. A household having no elderly member nor any member with a chronic illness was labelled as a "low health care need" household. A household having either (a) no elderly member but one or more member(s) with chronic illness, or (b) no member with chronic illness but one or more elderly members(s), or (c) one elderly member and one member with chronic illness was labelled as a "high health care need" household. A household with either (a) at least one elderly member and two or more members with chronic illness or (b) at least one member with chronic illness and two or more elderly members was labelled as a "very high health care need" household. This classification divided our total sample of 543 households into 181 low-need, 284 high-need and 78 very-high-need households. To validate our classification, we estimated the incidence of acute and chronic episodes (per 100 persons) and the number of out-patient visits and hospitalisations (per 100 households) for each need group. With a minor exception for the incidence of acute episodes, all other indicators show a steep rise as they move from low- to high- and from high- to very-high-need households, thereby validating our need-based classification.

#### Utilisation

Health care utilisation takes the form of out-patient visits or in-patient hospitalisations. Though the survey gathered detailed information about place, type of institution, and type and system of practitioner for all OP visits, we broadly divided OP visits into three categories: government, private informal and private formal/qualified. All hospitalisation episodes were broadly classified as government or private. Because health care services from government and private informal providers were expected to be less expensive, our classification of health care providers by type of institution was useful in controlling for the price/cost effect in our multivariate model.

#### Impoverishing effects of households' health care expenditure

Given the limitations of data in our case, we used two types of indicators to capture empirically the impoverishing effects of household health care expenditure. First, we identified households with high health care expenditure to see how those households were distributed across health care need categories. Second, we examined the various means by which households financed their health care expenditures. We used the concept of "high health care spending households" rather than the notion of catastrophic health care payments that is frequently used in the literature on equity of health care payments. We identified a household as having incurred high out-of-pocket expenditure on health care if its annual health care expenditure was high in comparison to those of other households within the same caste group. To define a high health care spending household (or "high-spending household", for brevity) in our context, we borrowed the concept of statistical outlier widely used in the literature on exploratory data analysis [33,34]. Suppose Q_1C _and Q_3C _are respectively the first and third quartiles of the distribution of per capita health expenditure of caste group C, then a high-spending or outlier household within caste group C is one for which the value of per capita health expenditure is greater than Q_3C_+k*(Q_3C_-Q_1C_), where k is a constant. Although most statistical analyses consider k = 1.5 for defining outliers, we can justifiably use two additional and lower values of k (0.5 and 1) in our context. Since Q_3C_-Q_1C _measures the spread of the middle 50% of per capita health expenditure values, k = 0.5 suggests that the range of the top 25% of expenditure should be half that of the middle 50%, and any expenditure value beyond that range should be considered an outlier. A less strict definition of outlier is suggested by k = 1, which allows the range of the top 25% expenditure values to equal the range of the middle 50% of expenditure values. In short, having three values for k, rather than one, gives us more flexibility by allowing us to define outlier households at different expenditure cut-off points.

## Results

### Sample characteristics

The sample households' socio-economic, demographic and health status characteristics are presented in Table 1. The table shows that caste is a good stratification variable for classifying households by social, economic and demographic characteristics. Compared to the FC, the socially backward castes (viz. Paniya, Other ST/SC, and OBC) have a higher share of female-headed households, a characteristic that generally adds to a household's vulnerability. Head-of-household's education clearly shows a caste gradient. The distribution of land, the most crucial economic asset, shows that the majority of the landless households are Paniya. The occupation profile also supports the Paniya households' status as the poorest. The households' annual per capita consumption expenditure (collected in the baseline survey) further confirms the caste gradient.

**Table 1:** Socio-economic and demographic characteristics of the sample households

	*Paniya*	*Other ST/SC*	*OBC*	*FC*	*Total*
*Sample households*	148	92	146	157	543
*Sample individuals*	841	461	845	778	2925
					
**% of female-headed HH**	18.9	20.8	19.9	15.9	18.6
**Education of HH head**					
No education	77.0	34.8	19.2	5.7	24.3
Primary	13.5	20.6	33.6	17.2	23.0
High school	9.5	44.6	45.9	72.0	50.5
Above high school	0.0	0.0	1.4	5.1	2.2
**Landholdings**					
No land	24.3	2.2	0.0	1.3	3.7
0.01-10 cents*	53.4	42.4	25.3	3.8	24.4
10.01-50 cents	13.5	20.8	42.5	19.1	26.8
50.01-100 cents	7.4	14.2	17.1	22.9	17.5
> 100 cents	1.4	20.5	15.1	52.9	27.7
**Occupation**					
Independent cultivator	2.7	27.0	30.1	60.5	36.9
Wage labourer	92.6	61.1	52.1	19.1	47.1
Govt/private service	0.0	5.4	6.2	11.5	7.1
Others	4.7	6.5	11.6	8.9	8.9
**Mean per capita consumption expenditure** [95% CI]	5083 [4465,5701]	6638 [6048,7229]	6668 [6083,7253]	8485 [7855,9115]	7109 [6771,7448]
**Mean household size** [95% CI]	5.7 [5.2,6.1]	5.0 [4.6,5.4]	5.8 [5.4,6.2]	5.0 [4.7,5.2]	5.3 [5.2,5.5]
**Households with elderly member (%)**	33.1	32.7	39.7	46.5	40.0
**Households with chronically ill member (%)**	28.4	61.9	62.3	66.9	59.9
**Level of health care need *(column-wise % distribution)***					
Low	49	26	29	27	30
High	43	67	59	47	54
Very high	8	6	12	27	16
**Share of private sector (%)**					
Total OP visits	30.8	52.8	66.3	71.7	65.1
Total hospitalisations	31.8	70.2	80.6	80.2	77.1

* One cent = 1/100th of an acre

Source: Wayanad baseline and panel surveys

### Caste-based inequality in per capita health expenditure

Like per capita consumption expenditure, per capita health expenditure across caste groups reaffirms the caste hierarchy (Table 2). The FC households show the highest per capita health expenditure, followed by the OBC, Other ST/SC and Paniya households, which show the lowest figure. Our need-based classification of households is validated by the per capita health expenditure of households with different levels of health care need. The per capita health expenditure is highest for very-high-need households, followed by high-need households and then low-need households.

**Table 2:** Per capita health expenditure (Rs) by household's level of health care need, type of illness episode, categorized by caste

	Paniya	Other ST/SC	OBC	FC	All castes
**Health care need**					
Low (N = 181)	35 [23, 43]	143 [97,163]	260 [221,281]	449 [338,512]	247 [192,278]
High (N = 284)	45 [27,56]	290 [266,305]	583 [428,687]	780 [666,854]	513 [415,576]
Very high (N = 78)	77 [34,89]	255 [232,257]	581 [550,593]	1085 [952,1168]	819 [808,824]
**Type of episode**					
Acute (N = 4408)	32 [24, 39]	162 [128,192]	258 [217,295]	414 [347,474]	263 [220,301]
Chronic (N = 430)	11 [3, 18]	89 [57,116]	242 [112,357]	387 [273,491]	233 [142,313]

**ALL**	**43** **[32,53]**	**251** **[221,277]**	**501** **[386,602]**	**801** **[695,897]**	**496** **[415,567]**

Note: Figures in the parentheses are sample sizes. Figures in the brackets are 95% confidence intervals.

Source: Wayanad panel surveys

**Figure 1:**
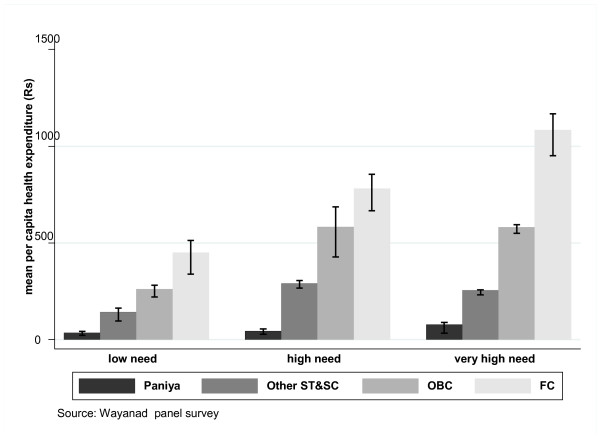
**Per capita health expenditure across household castes**.

Since it is to be expected that households' health care need, health care utilisation rate and choice of health care providers will vary, not all differences in per capita health expenditure across caste groups necessarily reflect inequality. For example, not all high-need households have a high health care utilisation rate, as they may face barriers in accessing health care. Further, a household's choice of health care providers may determine its health care expenditure, as OP visits to government and private informal health care providers cost less than those to formal private providers. If caste differences in per capita health expenditure were nothing unusual and did not necessarily reflect inequality faced by different castes in accessing necessary health care, one would expect to see similar patterns of caste differences among low-need, high-need and very-high-need households. Similarly, one would expect a similar pattern of caste differences for expenditure on acute and chronic illnesses. Figure 1 presents the per capita health expenditures for low-, high- and very-high-need households by caste. Although the gradual increase in per capita health expenditure is clearly evident, within each caste, for each group of households with comparable levels of health care need, the per capita health expenditure for each caste category does not show a similar rise when moving from low-need to high-need and from high-need to very-high-need. In the latter case, a steady rise in per capita health expenditure is clearly evident for FC households, but not for other caste groups, especially the Paniya. Irrespective of the level of health care need, a Paniya household, on average, spends less than 8% of what a need-comparable FC household spends per person. Further, it can be observed that a very-high-need household belonging to an Other ST/SC or OBC group spends a much lower percentage of what a need-comparable FC household spends on health care. This indicates that very-high-need households belonging to Other ST/SC and OBC households are not able to spend as much as they should on health care.

**Figure 2:**
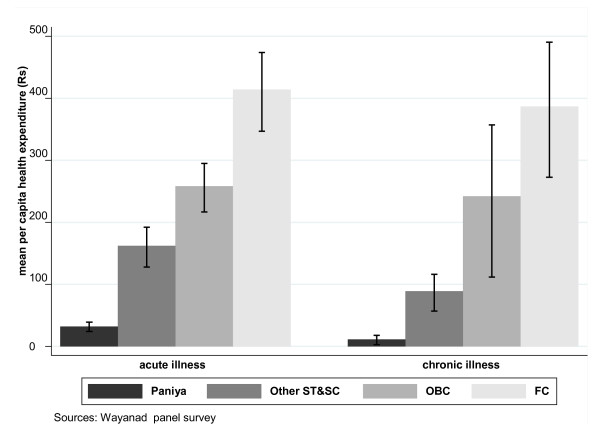
**Per capita health expenditure by type of episode**.

Figure 2 presents per capita expenditure on acute and chronic episodes by caste. For the population as a whole, per capita expenditure on acute episodes is slightly higher than expenditure on chronic episodes. The higher confidence interval of the latter compared to the former is an indication that households vary considerably from one to another in their per capita expenditure on chronic episodes compared to their expenditure on acute episodes (Table 2). Although the magnitude of difference varies, all castes show higher per capita expenditure on acute episodes than chronic episodes, but the difference is striking for the Paniya and Other ST/SC. Such a striking difference is not observed in the OBC class, which is socio-economically closer to the FC. This clearly points to a huge unmet need for chronic health care in the Paniya and Other ST/SC groups.

A closer look at Table 1 and Figures 1 and 2 therefore suggests that the observed overall caste gradient in health expenditure (Rs 43, Rs 251, Rs 501, Rs 801) is a product of compositional effect (composition of low-, high- and very-high-need households in each caste group) and a pure caste effect. Caste groups differ from each other in terms of their composition of low-, high- and very-high-need households (Table 1). Sorting household health expenditure by level and type of health care need shows that the caste gradient is stronger for high-need and very-high-need households (Figure 1) and for chronic health care need (Figure 2).

The greater inequality observed among high-need and very-high-need households probably indicates a violation of the vertical equity principle. We examined this issue more closely using a multivariate linear regression, as outlined in the *Framework *sub-section. The independent variables were household's caste, household's landholding, household's health care need, and other confounders such as sex of the household head, number of government and private OP visits, and number of hospitalisations. Household's landholding was included to capture the effects of a household's economic status on its out-of-pocket health expenditure that are not captured by the caste. Sex of the household head was included to examine whether having a female head places a household in an unfavourable position to allocate money to health care. It has been observed in the Indian context that female-headed households are more vulnerable to poverty due to gender bias in employment opportunities [35]. The numbers of OP visits and hospitalisations were included as confounders because per capita health expenditure is expected to be higher for households with a greater number of private OP visits and hospitalisations. Because a household's utilisation of health care is also expected to be dependent on its health care need, including both need and utilisation may put the model at risk of multicollinearity. However, we included the utilisation variables that capture both the number of OP visits and hospitalisation as well as the type of institution utilised (government/private) in order to capture the price effects of health care utilisation that have a strong bearing on the level of per capita health expenditure. To highlight the extent to which inclusion of utilisation variables made a difference in capturing caste-based inequality in health expenditure, we present both models: one without the utilisation variables (Model 1) and the other with them (Model 2). Because per capita health expenditure exhibited a positively skewed distribution, we made a log transformation to remove the skewness after adding Re 1 to the value of each household's per capita health expenditure.

Table 3 presents the results of the multivariate regression. Using FC as a reference caste, Paniya and Other ST/SC show negative coefficients that are significant in both models, indicating lower per capita health expenditure. It is important to observe that Other ST/SC, which is closer to OBC and FC in a number of characteristics, behaves like the Paniya. In both models, with reference to low-need households, both high-need and very-high-need households show significant increase in per capita expenditure. Neither the household's landholdings nor the sex of the household head is significant in the models, which is an indication that they do not play a role in lowering health expenditure. In Model 2, more OP visits to government or informal care providers do not significantly increase per capita health expenditure, but OP visits to private facilities result in a significant increase. A household's episodes of hospitalisation, whether public or private, are associated with a significant increase in its per capita health expenditure. In a nutshell, with reference to FC, the Paniya and Other ST/SC households spend less on health care per capita, even when we take into account the effects of the household's health care need and its volume of government and private health care utilisation.

**Table 3:** Regression results Dependent variable = Ln (per capita health expenditure+1)

	*Model 1*		*Model 2*	
	coefficient	P > t	coefficient	P > t
**Constant**	5.527	0.000	4.531	0.000
**Caste (Ref: FC)**				
Paniya	-3.480	0.000	-2.860	0.000
Other ST/SC	-0.963	0.000	-0.621	0.000
OBC	-0.128	0.421	-0.174	0.198
**Household's landholdings (Ref: > 100 cents)**				
0-10 cents	-0.409	0.034	-0.040	0.810
11-50.00 cents	-0.181	0.300	-0.053	0.719
50.01-100.00 cents	-0.320	0.085	-0.062	0.695
**Need (Ref: low)**				
High	0.720	0.000	0.399	0.001
very high	1.210	0.000	0.477	0.009
**Sex of household head (Ref: Male)**				
Female	0.063	0.684	0.210	0.110
**Utilisation of health care**				
OP visits: government & informal	--	--	0.004	0.211
OP visits: private	--	--	0.072	0.000
Hospitalisation: government	--	--	0.514	0.000
Hospitalisation: private	--	--	0.534	0.000
***R-squared***	0.4741		0.6273	
***Adjusted R-squared***	0.4652		0.6182	

Note: **Model 1**: ln (per capita health expenditure + Re 1) = f (caste, landholdings, health care need, sex of household head); **Model 2**: ln (per capita health expenditure + Re 1) = f (caste, landholdings, health care need, sex of household head; number of government and private OP visits and hospitalisations)

Source: Wayanad baseline and panel surveys

**Figure 3:**
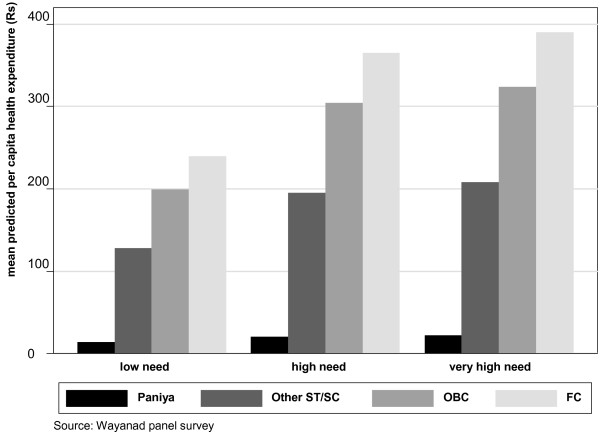
**Predicted per capita health expenditure**.

To shed further light on caste-related inequality in per capita health expenditure, we used the estimated regression equation of Model 2 to predict average values of per capita health expenditure incurred by the four caste groups for each level of health care need (viz. low, high and very high). Because we used household's landholdings, sex of the household head, number of OP visits and hospitalisations as confounders in the regression, we estimated the predicted values of health expenditure at the mean values of the confounding variables. The predicted values, presented in Figure 3, highlight certain aspects of inequality that were uncovered in Figure 1. First, moving from low-need to very-high-need households, both FC and Other ST/SC caste groups are able to systematically increase per capita monetary allocation on health care - a pattern which is not observed for the Paniya and the OBC caste groups. Second, there is no difference between Paniya low-need and high-need households in per capita health expenditure, while the very-high-need *Paniya *households' expenditure is marginally higher.

### High-spending households and their economic burden

Table 4 presents the cut-off level of per capita health expenditure for identifying high-spending households, the percentage of high-spending households and the distribution of high-spending households across low-, high- and very-high-need categories. Looking at the cut-off levels of health expenditure for the four caste groups at three different values of k, two points are clear. First, at any given value of k, the health expenditure cut-off levels follow the caste hierarchy, that is, the cut-off is lowest for the Paniya and highest for the FC. Second, for any given caste group, the health expenditure cut-off level is higher for higher values of k.

**Table 4:** Households with high health expenditure and their distribution across health care need categories

	Health expenditure cut-off (Rs)	Outlier households (%)	Distribution of outlier households by levels of households' health care need (%) [low, high, very high]
**K = 0.5**			
Paniya	70.5	18.2	[37, 44, 19]
Other ST/SC	555	14.1	[8, 85, 8]
OBC	866.5	15.8	[4, 83, 13]
FC	1508.5	14.0	[18, 46, 36]
All castes	940	15.6	[11, 54, 34]
**K = 1**			
Paniya	94	14.9	[32, 50, 18]
Other ST/SC	718	6.5	[17, 83, 0]
OBC	1105	10.3	[7, 80, 13]
FC	1928	7.6	[8, 50, 42]
All castes	1220	10.1	[11, 58, 31]
**K = 1.5**			
Paniya	117.5	12.8	[37, 42, 21]
Other ST/SC	881	2.2	[0, 100, 0]
OBC	1343.5	7.5	[0, 91, 9]
FC	2347.5	5.1	[0, 63, 37]
All castes	1500	7.5	[12, 61, 27]

Source: Wayanad baseline and panel surveys

Although the Paniya caste group spends much less on health care per capita compared to households from other caste groups, the distribution of per capita health expenditure within the Paniya caste shows that a higher percentage of Paniya households incur large expenditure on health care. However, Other ST/SC shows the lowest percentage of high-spending households (except at k = 0.5, where its expenditure is marginally higher than that of the FC). Furthermore, most of the high-spending households belong to either the high-need or very-high-need categories. At all three values of k, more than 85% of the households belong to the high-need or very-high-need categories. Though this pattern is observed in the Other ST/SC, OBC and FC caste groups, the Paniya group seems to be different, as 32%-37% of high-spending Paniya households have low health care need. In other words, a Paniya household may spend relatively more on health care irrespective of its level of health care need - a pattern not observed in other caste groups.

The choice of health care provider seems to affect a household's likelihood of impoverishment due to out-of-pocket health expenditure. Whereas the number of government (or informal) OP visits has no significant effect on the level of a household's out-of-pocket health expenditure, private OP visits and hospitalisations (government or private) have significant positive effects on the level of out-of-pocket health expenditure (Table 3). The higher values of the coefficient of hospitalisation at government or private facilities clearly indicate that a household is most likely to fall into the high-spending category if it experiences an episode of hospitalisation, irrespective of the type of hospital - government or private - utilised. This hypothesis is also corroborated by the distribution of OP visits and hospitalisations by type of health care provider for households that fall under the category of high-spending and for those that do not. Among the households that did not incur high out-of-pocket health care expenditure, 42% of OP visits and 69% of hospitalisations were in private facilities. However, high-spending households showed much higher percentages of OP visits (55%) and hospitalisations (92%) at private facilities. In other words, households with higher numbers of OP visits to private providers and hospitalisations at private facilities are more likely to be high-spending.

The distribution of households' out-of-pocket health care expenditure by sources of financing highlights an important dimension of the hardship faced by different caste groups in mobilising monetary resources to meet health care expenditure (Table 5). Available cash or current income contributes 65% of households' total out-of-pocket health expenditure, followed by loans from friends, self-help groups or money lenders (24%), savings including selling of food stocks (8%), and donations from friends or relatives (3%). Two questions seem important in this context: (a) Is the distribution of total out-of-pocket health care expenditure by sources of financing similar for all caste groups? (b) Is the pattern of financing health care expenditure similar for OP visits and for hospitalisations? Table 5 shows that Paniya and OBC households depend more on loans and donations for meeting total health expenditure than do FC households. It is worth noting that in spite of Paniya households' having very low per capita health expenditure, 31% of their out-of-pocket health expenditure is financed by loans and donations. Though Other ST/SC households finance more than three-quarters (77%) of their total out-of-pocket health expenditure from available cash and savings, this probably entails compromise in their consumption of other basic necessities. Despite spending only one-third of total out-of-pocket expenditure on hospitalisation, the households depend on loans and donations more for hospitalisation than for OP visits. Whereas only 21% of households' out-of-pocket expenditure on OP visits comes from loans (19%) and donations (2%), 43% of their expenditure on hospitalisation comes from loans (40%) and donations (3%). Patterns of financing hospitalisation expenditure reveal greater vulnerability in all backward castes as compared to FC. The percentages of hospitalisation expenditure financed by loans and donations from friends and/or relatives for the Paniya, Other ST/SC, OBC and FC households are 48, 45, 45 and 42 respectively. There is no evidence to suggest that backward caste groups depend more on loans to finance expenditure on OP visits compared to the FC group. Despite Paniya households' having a very low expenditure level, one-quarter of their total household out-of-pocket expenditure on OP visits is financed by loans (23%) and donations (2%). This is a clear indication of their vulnerability in meeting out-of-pocket health expenditure. It is interesting to observe that Other ST/SC households mobilise a large portion of their health expenditure (especially hospitalisation expenditure) from donations. This is an indication of a much higher level of social networking among these tribe and caste groups.

**Table 5:** Distribution of households' out-of-pocket health expenditure by sources of finance

Caste	Per capita expenditure (Rs)	Distribution of out-of-pocket health expenditure by sources of finance (%)			
		**Cash^1^**	**Savings^2^**	**Donations^3^**	**Loans^4^**

Total health expenditure*					
Paniya	43	62	7	3	28
Other ST/SC	251	71	6	5	19
OBC	501	63	6	4	28
FC	801	66	10	1	22
All castes	496	65	8	3	24
***Expenditure on OP visits***					
Paniya	31 [25,36]	70	4	2	23
Other ST/SC	204 [186,216]	77	5	1	17
OBC	353 [324,375]	68	6	4	22
FC	641 [579,687]	71	10	2	18
All castes	379 [347,403]	70	8	2	19
***Expenditure on hospitalisation***					
Paniya	12 [4,17]	37	14	7	41
Other ST/SC	47 [25,62]	45	10	19	26
OBC	148 [26,237]	50	5	4	41
FC	160 [110,198]	49	9	1	41
All castes	117 [51,164]	49	7	3	40

Notes: 1 - Available cash mostly from currently income; 2 - Includes savings as well as selling of food stocks; 3 - Donations from relatives or friends; 4 - Includes loans from friends, self-help groups or money lenders; Figures in the brackets show 95% CIs.

Source: Wayanad baseline and panel surveys.

## Discussion

Looking at various socio-economic indicators reveals a caste hierarchy with FC at the top of the ladder, followed by OBC and Other ST/SC, and Paniya at the bottom. The per capita health expenditures reported by these caste groups accord with this caste hierarchy, with FC and Paniya households' health care expenditure being very high and very low, respectively. In its last two health rounds, India's National Sample Survey Organisation observed people's high dependence on private providers, which has increased over the years, even among the poor and marginalised [17-19]. In a context where households depend on private health care providers to a great extent for meeting their health care needs, differences in per capita health expenditure indicate inequality in access to quality health care. Moreover, the fact that poor households spend less on health care does not mean their indirect costs of illness are low. Even if poor households spend significantly less on health care, they incur a higher proportion of health-related loss of income than do other non-poor groups [36].

Our analysis shows that caste differences in per capita health expenditure are not similar for households with different levels of health care need, and there is an indication that very-high-need households belonging to the Paniya, Other ST/SC and OBC caste groups do not have the means to cover what they are required to spend for health care. On average, households spend slightly more annually on acute episodes than on chronic episodes, but unlike their expenditure on acute episodes, households in each caste group vary considerably among themselves in per capita expenditure on chronic episodes. Compared to the FC and OBC caste groups, the acute-chronic difference in per capita expenditure is greater among the Other ST/SC and Paniya caste groups. From the per capita expenditure figures, there appears to be a huge unmet need for chronic health care in Paniya and Other ST/SC households, though Paniya households report less need for chronic health care. Most studies on health financing in the Indian context have recommended introducing or scaling up social health insurance as the only remedy for improving access to health care for the poor and marginalised [37,38]. Our findings question the effectiveness of these remedial measures, as many of the existing health insurance packages pay very little attention to chronic health care need, especially in the elderly population. It has been pointed out that very little analysis was done before various social health insurance schemes were proposed as remedial measures to solve problems of health care access for the poor in the Indian context [39]. Many of these recommendations either ignored or downplayed the immediate need to strengthen supply side factors, i.e., health care infrastructure and manpower, including in the government health sector. The lower need for chronic health care expressed by the Paniya caste groups also calls for further scrutiny.

The multivariate analysis shows that Paniya and Other ST/SC households spend significantly less on health per capita than do FC households, even taking into account the effects of levels of health care need, household's landholding, vulnerability of female-headed households, and volume and type of health care utilisation. The insignificance of the variable 'landholding' is an indication that the social variable 'caste' adequately captures the effect of socio-economic status on per capita household expenditure. Though higher utilisation of government and private informal OP services makes no significant positive impact on per capita health expenditure, private OP visits and hospitalisations (government or private) do produce a significant increase. This is in line with an earlier study in Kerala which found that, even in government hospitals, households spent significant amounts of money on buying services outside the hospital [36].

It has generally been observed that health care payments and financial burden (payment share) increase with an increase in ability to pay [20]. Therefore, owing to the steep income gradient, FC households would be expected to have higher health care payments and a greater financial burden than other caste groups. Studies have shown that it is not only the better-off but also poor households that can be at the risk of large health care payments [40,41]. Our analysis also points to the vulnerability of backward caste groups, especially the Paniya, when exposed to relatively high health expenditure. While the per capita health expenditure of the Paniya caste group is lowest, a higher proportion of Paniya households incur relatively large expenditure compared to the other caste groups. This is an indication that not all Paniya households are in a position to take advantage of free or nearly free public health care. The Paniya's low utilisation of government health care suggests that unless steps are taken to remove the social barriers to health care access faced by marginalised populations, bringing them under social insurance may not improve their access to health care. In contrast to the Other ST/SC, OBC and FC groups, in which households that spend relatively large amounts on health care have either high or very high needs, a large percentage of Paniya households with low health care need spend relatively large amounts on health care. This has an important policy implication, which is that while high-need and very-high-need households belonging to Other ST/SC, OBC and FC caste groups need financial protection, *all *Paniya households need universal protection.

In comparison to the FC group, the Paniya and OBC caste groups depend more on loans and donations for meeting total health expenditure. The households' patterns of financing expenditure for OP visits and hospitalisations were found to be different. It has been found in the Indian context that out-patient care is more impoverishing than in-patient care in urban and rural areas [6]. Other empirical findings suggest that high health expenditure for a household is not usually the result of one single disastrous event such as hospitalisation, but rather a series of events [42,43]. Our analysis in Kerala showed that, per year, a household's average expenditure on hospitalisation was less than one-third of what it spent on OP visits. This is significantly higher than what has been observed elsewhere in India [43,44]. It has been found in other parts of India that low expenditure on hospitalisation is due to low utilisation of hospital care. The concentration of hospitals mostly in urban areas and district headquarters is a barrier to access for rural populations, resulting ultimately in their low utilisation of hospital care [44]. However, a larger hospitalisation component in total health care expenditure in Kerala is not unusual, since Kerala also reports a much higher incidence of hospitalisation compared to other Indian states [17,18]. Contrary to other studies [42,43], we found that hospitalisation expenditure has more impoverishing effects on households, as evidenced by our analysis of the distribution of households' out-of-pocket expenditure by sources of finance. The households depend more on loans and donations to meet hospitalisation expenditure than they do to meet expenditure on OP visits. The patterns of financing hospitalisation expenditure clearly show the Paniya, Other ST/SC and OBC households to be more vulnerable than FC households. This is most likely due to the unpredictable nature and large amount of the hospitalisation expenses. Other studies have found that households' preference for private health care, economic status, utilisation of modern medical care, presence of ill elderly member(s), presence of member(s) with chronic illness and incidence of hospitalisation are key determinants of high health expenditure [40,41].

## Conclusion

The per capita health expenditures reported by the four caste groups accord with their status in the caste hierarchy, with FC spending the most and Paniya spending the least. In a context with almost no health insurance coverage, inadequate public provision of health care and increasing reliance on private providers, the differences in per capita health expenditure by various caste groups are a clear reflection of their unequal access to quality health care. Multivariate analysis also confirmed this caste-related inequality after controlling for health care need and influential confounders. Among the Paniya, Other ST/SC and OBC caste groups, households with high health care need and chronic health care need were found to suffer more due to this inequality. This calls for a fresh look into the widespread belief that Kerala manifests less social inequality in access to health care. The steeper caste gradients for high-need and very-high-need households and for chronic care episodes are clear indications of the lower castes' severe deprivation in accessing care, especially in situations of high and chronic health care need. The Paniya caste group clearly suffers most.

Our analysis shows that households with high health care needs are at higher risk of incurring large expenditures on health care. Among the lower castes, the Paniya, in spite of their low per capita health expenditure, seem to be the most vulnerable at all levels of health care need. Therefore, apart from fully protecting the scheduled tribe households, especially the Paniya tribes, against impoverishing health expenditure, there is a strong need to protect all households that have high potential need for health care, especially households with members who are elderly and/or suffering from chronic illness. Validation of our definition of health care need confirmed that high-need and very-high-need households have a higher disease burden, expressed in their utilisation of OP visits and hospitalisation for acute and chronic episodes. As evidenced by the sources of financing, hospitalisation expenses seem to have the most impoverishing impacts on households, especially on Paniya, Other ST/SC and OBC households. Special emphasis must therefore be given to funding hospitalisation, as this expenditure puts households more at risk in terms of mobilising monetary resources than does expenditure on OP visits. However, designing protection instruments requires a deeper understanding of how the uncovered financial burden of expenditure on OP and hospitalisation produces negative consequences and of the relative magnitude of this burden on households.

With regard to the scope of our study, three limitations should be noted. First, though we attempted to analyse caste-based inequality in out-of-pocket health expenditure, we did not capture inequality related to gender and age within caste groups. Other studies have found these inequalities to be more present in marginalised and vulnerable populations. Second, we did not attempt to measure empirically the magnitude of the financial burden of out-of-pocket health expenditure at the household level. Rather, by identifying high-spending households across caste groups and how households belonging to different caste groups financed their out-patient and hospitalisation expenditures, we provided only indirect evidence of financial burden. Third, to shed light on households' coping mechanisms for health care expenditure, we showed only how households belonging to different caste groups mobilised money from various sources. While it would have been illuminating to be able to analyse how high expenditure on health affected households' consumption of basic necessities, unfortunately the limitations of the present data set did not permit such analysis.

## List of abbreviations used

FC: Forward Castes; HH: household; OBC: Other Backward Castes; OP: out-patient; Re/Rs: Indian currency Rupee/Rupees; SC: Scheduled Castes; ST: Scheduled Tribes.

## Competing interests

The authors declare that they have no competing interests.

## Authors' contributions

SM conceived the study problems with inputs from SH, performed the statistical analysis and wrote the initial draft of the manuscript. SH and DN are principal investigators of the larger study, led its design, coordinated data collection, advised on statistical analysis and assisted in revision of the manuscript. All authors read and approved the final manuscript.
